# Full-scale simulations to improve disaster preparedness in hospital pharmacies

**DOI:** 10.1186/s12913-022-08230-9

**Published:** 2022-07-02

**Authors:** Laurence Schumacher, Salim Senhaji, Birgit Andrea Gartner, Laurent Carrez, Arnaud Dupuis, Pascal Bonnabry, Nicolas Widmer

**Affiliations:** 1 Specialised Centre for Emergency and Disaster Pharmacy, Institute of Pharmaceutical Sciences of Western Switzerland, School of Pharmaceutical Sciences, University of Geneva, Geneva, Switzerland; 2 Pharmacy of the Eastern Vaud Hospitals, Rennaz, Switzerland; 3 Pharmacy, Geneva University Hospitals, Geneva, Switzerland; 4 Emergency Department, Geneva University Hospitals, Geneva, Switzerland; 5 Service of Pharmacy, Lausanne University Hospital, Lausanne, Switzerland; 6 Surgery Department, Geneva University Hospitals, Geneva, Switzerland; 7 Specialised Centre for War and Disaster Surgery, Geneva University Hospitals, Geneva, Switzerland

**Keywords:** Disaster planning, Full-scale exercises, Simulation, Pharmacy service, Hospital

## Abstract

**Purpose:**

Assess whether full-scale simulation exercises improved hospital pharmacies’ disaster preparedness.

**Methods:**

Swiss hospital pharmacies performed successive full-scale simulation exercises at least four months apart. An interprofessional team created two scenarios, each representing credible regional-scale disasters involving approximately fifty casualties (a major road accident and a terrorist attack). Four exercise assessors used appraisal forms to evaluate participants’ actions and responses during the simulation (rating them using five-point Likert scales).

**Results:**

Four hospital pharmacies performed two full-scale simulation exercises each. Differences between exercises one and two were observed. On average, the four hospitals accomplished 69% ± 6% of the actions expected of them during exercise one. The mean rate of expected actions accomplished increased to 84% ± 7% (*p* < 0.005) during exercise two. Moreover, the average quality of actions improved from 3.0/5 to 3.6/5 (*p* = 0.01), and the time required to gather a crisis management team drastically decreased between simulations (from 23 to 5 min). The main challenges were communication (reformulation) and crisis management. Simulation exercise number one resulted in three hospital pharmacies creating disaster action plans and the fourth improving its already existing plan.

**Conclusion:**

This study highlighted the value of carrying out full-scale disaster simulations for hospital pharmacies as they improved overall institutional preparedness and increased staff awareness. The number of expected actions accomplished increased significantly. In the future, large-scale studies and concept dissemination are warranted.

## Introduction

Disaster preparedness is encouraged for all healthcare professionals, including hospital pharmacists [1–8]. Gaps in preparedness could even magnify the adverse effects of an unexpected disaster [9], and this is why governments and guidelines actively encourage hospitals to improve their preparedness [1–7]. For instance, the Canadian Association of Emergency Physicians recommends conducting drills to test, train, and educate all healthcare professionals on disaster preparedness [10]. Pharmacists’ efforts to improve their preparedness for major disasters have direct impacts on their potential to save lives [11]. Unfortunately, there are few descriptions of hospital pharmacies’ levels of preparedness in the literature [11]. One recent survey of European hospital pharmacies revealed a widespread lack of disaster preparedness, with only a few organizing exercises to improve their capabilities in this area [12]. The current COVID-19 pandemic has also shown that hospital pharmacies are highly involved in and greatly affected by health crises yet [13–19], suggesting that preparedness would be a significant benefit [20].

Simulations are pedagogical methods used to imitate real-world processes or systems to safely train healthcare students or professionals in a very practical way. Two meta-analyses have shown that simulation exercises have positive effects on knowledge [21, 22]. This active, innovative method has the advantage of putting healthcare staff’s knowledge and coordination directly into practice [4, 23] using a simulated real-world disaster to assess their responses [23, 24]. There are many means of conducting simulation exercises, including tabletop exercises, full-scale field exercises, simulators, augmented reality, and functional real-time exercises [24]. Moreover, simulation can be used for both individual and team training [25]. This method is often used to improve knowledge in low-frequency but high-impact disaster situations. Simulation can also be used to prepare healthcare professionals for new situations [10, 26]. However, most studies have focused on physicians [27], paramedics [27, 28], or nurses and not pharmacists [27, 29]. Nonetheless, additional studies are needed to improve knowledge in this field, to facilitate assessments of healthcare professionals’ preparedness, and to determine institutional levels of preparedness for different types of disasters [4, 30]. According to the WHO, full-scale exercise is “an interactive activity that includes real-time conduction and actual deployment of resources in response to a fully simulated emergency” [24]. Furthermore, a drill is “an operational activity for maintaining and developing skills in a single-response procedure” [24, 31]. Several drill methods exist for testing disaster plans and improving teams’ skills in healthcare settings. Whatever the type of intervention, such drills have shown positive effects on disaster preparedness when they properly represented real-life events [25, 32–34]. To the best of our knowledge, there are no published studies evaluating the use of full-scale simulation exercises to drill hospital pharmacies in the management of major incidents or disasters.

The World Health Organization (WHO) has published recommendations on practicing emergency exercises in hospitals [24], and the French National Health Authority (*Haute Autorité de Santé*; HAS) has published guidelines on good practices in healthcare simulation in general [31]. The latter recommended structuring simulation exercises in three phases: briefing, playing out the scenario, and debriefing [31]. According to the HAS, a short briefing should be scheduled at the beginning of an exercise to explain the context and to get participants in the mood and motivate them [24, 31]. Script scenarios should be written to meet educational objectives [31, 35]. The literature review identified four key points to developing a productive simulation: planning, staff engagement, realism, and debriefing [33, 34, 36].

The present study aimed to assess whether full-scale simulation exercises improved hospital pharmacies’ disaster preparedness.

## Method

The eight chief hospital pharmacists making up the Group of Swiss French-speaking Hospital Pharmacists (*Groupement des Pharmaciens Hospitaliers Romands*; GPHR) were personally invited to participate in our prospective multicenter study (inclusion criterion). Those chief hospital pharmacists not wishing to or unavailable to participate in both exercises were excluded. Those who volunteered were asked to provide demographic data by answering 15 questions via the SurveyMonkey® web-based survey platform (SurveyMonkey, San Mateo, CA, USA; http://www.surveymonkey.com/). Each participating chief pharmacist readied disaster preparedness simulations for their facilities in secret. They informed their heads of units or departments on the morning of the simulation only to enable them to take every measure needed to ensure the continuity of routine activities and patient safety in parallel with the simulation. Chief pharmacists participated in the simulations as exercise assessors and disaster management response was led by their deputies. Pharmacy staff members were invited to a briefing a few minutes before the beginning of the simulation, thus reproducing the spontaneous onset of a real disaster: the drill’s objectives, the organization of a simulation, and how it would progress were explained.

Four chief pharmacists agreed to participate in the study. Two pharmacies (A and B) belonged to university hospitals with more than 1,000 beds. The two other pharmacies were responsible for between 200 and 1,000 beds. None of the four pharmacies had experienced a major incident or disaster in the past five years. Only one hospital pharmacy (D) had disaster standard operating procedures (SOPs) in place before the study began, and it had previously organized a short training session on disaster preparedness (when on-boarding new employees) and a table-top exercise on hospital disaster management. The three other hospital pharmacies had no SOPs because disaster preparedness was not considered a priority.

This study consisted of each participating hospital pharmacy running two different full-scale simulation exercises. The first exercise was used to measure hospital pharmacies’ initial levels of response and preparedness (to provide baseline data and identify the priority improvements needed). The second exercise was organized to take place at least four months later, using a second scenario and comparing the efficiency and effectiveness of the responses to those of the first exercise. The two same scenarios were used in each participating hospital pharmacy. Data collection ran from August 2018 to August 2020.

Every full-scale exercise lasted three to four hours and was structured according to the HAS [31] and WHO [24] health simulation guidelines. They were finished with a “hot debriefing” for all those directly involved in the simulation. This debriefing was structured in three phases as per the guidelines: description of the facts (brief summary of the scenario and a participant-led description of their perceptions), analysis (participants identification of the positive and negative effects of their actions), and synthesis (summary and feedback by the exercise assessors and identification of the improvements needed in the pharmacy). Subsequently, a written report summarizing the exercise assessors’ comments was used as feedback in a “cold debriefing” at a later date. Finally, each pharmacy used its report to decide on future actions and work on an improvement plan.

The research protocol, which involved no collection of patient data, was presented to the Canton of Geneva’s Research Ethics Committee, which confirmed that the project was not subject to their authorization according to Swiss law.

### Scenario

The two scenarios, each representing a credible regional disaster, were created by a team of pharmacists and physicians. The simulated disasters had to require more than the pharmacy’s usual capacities. The first scenario described a road traffic accident involving a bus, a truck, and other vehicles on a high-speed highway close to the hospital. Approximately 50 people were portrayed as injured, including contaminations with chemicals and carbon monoxide. The second scenario referred to a multi-site terrorist attack developing in three major phases: a poison gas (sarin) attack in the city’s main railway station, a shooting in the market area close to the hospital, and a secondary bomb attack also in the city’s main railway station. In both scenarios, the hospital’s emergency, anesthesia, and intensive care departments staff needed large amounts of drugs, including antidotes, and urgent pharmaceutical assistance. The scenarios were composed of 20 and 24 steps, respectively. The two scenarios will be freely available in French on the Specialised Centre for Emergency and Disaster Pharmacy’s website (http://www.disaster-pharmacy.ch/).

### Evaluation

Evaluations were done simultaneously by four exercise assessors, with each assessing one of four areas of activity by direct observation: communication, disaster management, logistical activities, and pharmaceutical assistance (coupled with the pharmaceutical manufacturing activities for practical reasons). The four exercise assessors were the chief pharmacist and an emergency physician from the participating hospital, plus two experts in health simulations and/or quality assessment (i.e., a physician, pharmacist, or quality assurance officer, depending on the hospital pharmacy). The Situation, Background, Assessment, Recommendation and Request Summary model—the SBARS model—was used to evaluate specific disaster communication between the different actors. SBARS is a structured, systematic communications method, part of the TeamSTEPPS set of teamwork tools [37, 38].

Exercise assessors evaluated participants’ simulation responses and actions in their specific areas of expertise using the Harvard School of Public Health's Emergency Exercise Evaluation Toolkit [37]. This proposes qualitatively and quantitatively measurable response elements based on objective data. Expected actions at each step in the scenario were listed for the exercise assessors, each action was evaluated as “performed or not”, and the quality of the performance was scored on a Likert scale of 1 to 5 (1 = not at all satisfactory, 2 = unsatisfactory, 3 = satisfactory, 4 = good, and 5 = excellent). This was a slight simplification of the Harvard assessment instrument, which scores actions from 1 to 10. For actions cutting across more than one domain, an average score was calculated from the evaluations of several exercise assessors. Actions evaluated not to have been performed were scored 1. Exercise assessors could leave comments related to each action or to the exercise as a whole in the free text fields provided. Finally, after the simulation, assessors were able to make suggestions for improvements based on their observations or expert opinion, and they were asked to highlight three strong points among the pharmacy staff’s actions, as well as three weak points requiring improvement. The key recommendations and areas for improvement identified in these data were used to structure the debriefing and the final written report [37].

### Data analysis

All the data collected by the exercise assessors were compiled for statistical analysis. Raw data were exported into Microsoft Excel® 2013 software (Microsoft Corporation, Redmond, WA, USA), which was then used to calculate descriptive statistics. T-tests for “before and after” comparisons were carried out using STATA® (version 14.0, StataCorp, College Station, TX, USA), and *p*-values less than 0.05 were considered statistically significant.

## Results

**Fig. 1:**
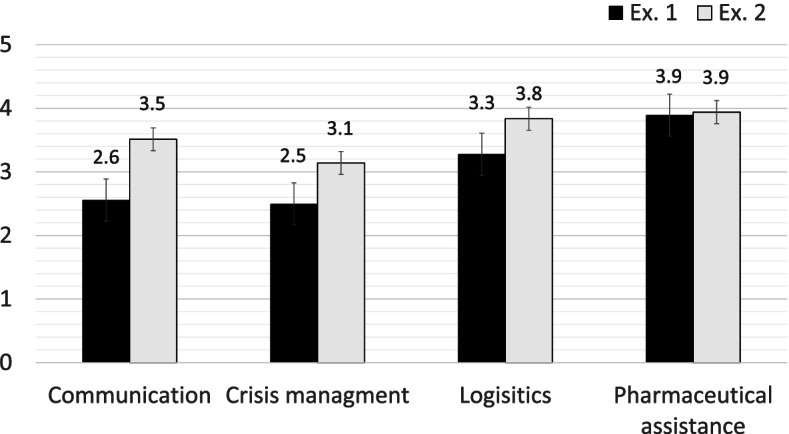
Quality o f the actions accomplished by type of activities evaluated (No = 1 and Yes = 1 to 5; if the expected action was not performed, it was given a score of 1)

In each hospital pharmacy, scenario one (a road traffic accident) was played out during the first simulation, and scenario two (a terrorist attack) was played out second. The mean rate of required actions accomplished increased from 69% ± 6% during the first exercise to 84% ± 7% (*p*-*value* < 0.005) during the second. The mean quality of all actions increased from 3.0/5 in the first exercise to 3.6/5 in the second (*p*-*value* = 0.01). The mean time required for the hospital pharmacy leader to gather their disaster management team decreased from 23 min (min. 5, max. disaster management team not set up) in the first simulation to 5 min (min. 4, max. 5) in the second. Table 1 shows each pharmacy’s results for both full-scale simulation exercises. Figure 1 shows the mean quality of the actions taken as estimated by the exercise assessors for the first and second exercises in each of the four activity types.

**Table 1:** Results of the full-scale exercise

	Pharmacy results								Averages		
	A		B		C		D		1	2	T-test *p* value
Scenario n°	1	2	1	2	1	2	1	2			
Number of scenario items	20	24	20	24	20	24	20	24			
Time between simulations [months]	-	6	-	8	-	14	-	4	-	8	
Percentage of actions accomplished [%]	62	75	66	83	76	91	71	85	69	84	< 0.005
Average quality of actions (1 to 5)	2.7	3.4	2.8	3.5	3.3	3.7	3.3	3.7	3.0	3.6	0.01
Time to gather disaster management team [min]	50	5	∞	4	15	5	5	5	23.0	5.0	
Duration of simulation [min]	210	195	200	165	205	180	210	190	206	183	
Disaster standard operating procedures (SOPs)	No	Yes	No	Yes	No	Yes	Yes	Yes			

No formal disaster management structure set up established at all during the full-scale simulation

Hospital pharmacy staff reacted very positively to both the exercises and took part fully and enthusiastically in the spirit of the simulation. Participants underlined how useful this training was, and all the chief pharmacists described the simulation as “very useful”. Table 2 summarizes the areas requiring improvements, as identified by the exercise assessors during the simulations and the improvements they observed during the second exercises.

**Table 2:** Gaps and improvements identified by the assessors during the exercises

	**Gaps identified during exercises**		**Improvements observed in the second exercises**	**Additional improvements suggested by the assessors**
	**First**	**Second**		
**Disaster standard operating procedures (SOPs)**	●Only one pharmacy had SOPs ●Hospital management’s difficulties in informing the pharmacy of hospital SOPs (except for the pharmacy with SOP) ●Lack of hierarchical disaster management structure	●Lack of enough tested informational and situational dashboards	●Presence of SOPs in every pharmacy ●SOPs consulted early ●Easier, more comprehensive triggering of SOPs ●Use of dashboards	●Train staff to use SOPs and dashboards
**Allocation of roles**	●No clear disaster management leader ●Spontaneous but uncoordinated allocation of tasks ●Lack of work delegation ●Lack of anticipation ●Only one pharmacy had action card, resulting in disorganized task attribution	●Only one pharmacy had actions cards ●Lack of delegation of specific tasks ●Difficulty designating or identifying a manager for each process/department ●Poor optimization of human resources	●Leader more clearly identifiable	●Create actions cards
**Management**	●In pharmacies without SOPs, management committees were set up slowly or not at all ●Pharmacy heads’s mix of management roles and operational tasks ●Lack of knowledge about the concept of disaster management, and no SOPs ●Lack of organization and rhythm in disaster management (no meeting points, …) ●Tendency to include too many people in the disaster management team ●No feedback requested on the evolution of the tasks delegated	●Inability to maintain overall situational awareness of the pharmacy ●Inability to manage and distribute tasks and collect feedback	●Management teams established rapidly	●Organize a rhythm to management via regular, scheduled meeting points
**Responses by different hospital pharmacy units**	●Poor redistribution of human resources ●No identification of leaders for the most affected processes or departments ●Lack of overall coordination and management ●Lack of separation between disaster-related and routine work flows	●No identification of leaders for the most affected processes or departments	●Improved separation of flow of disaster and routine requests to the pharmacy	●Identify leaders for each of the most affected processes or departments
**Communication**	●No structured communication (no reformulation) ●Lack of pharmacy feedback on actions requested by other hospital departments ●Poor, unstructured communication both up and down the hierarchy, between management and staff and between different pharmacy departments ●Under-utilization of the means of communication available	●Insufficient targeted communication with staff ●No acknowledgement of messages received (reformulation to demonstrate comprehension)	●Improved general communication ●Improved communication of SOPs to all employees	●Train and practice structured communication in routine practice (especially restating reformulating requests for action to demonstrate comprehension) ●Make sure to have regular status meeting points with a representative from each pharmacy department

After their first exercise, the three hospital pharmacies without a disaster plan each developed one before the second simulation, which nobody other than their chief pharmacist knew would happen. The pharmacy that already had disaster SOPs worked to improve them after the first simulation.

## Discussion

We developed a concept of full-scale disaster simulation exercises and tested it in several pilot hospital pharmacies. The two different scenarios used were comparable in terms of the similar numbers of steps occurring as the scenarios developed and the level of impact they would have on hospital pharmacies. The hospital pharmacy teams greatly appreciated all the full-scale simulation exercises, and all the chief pharmacists responded that this type of training was very useful for them, their staff, and their institution. These sentiments are also found in the literature, which reports that teams of healthcare professionals generally receive simulations well, describing them as a useful way to improve their practice and knowledge [10, 34]. In addition, these full-scale simulations highlighted the importance of pharmacy in disaster response through two realistic scenarios validated by physicians. It is also worth noting that the importance of pharmacies and pharmacy teams during disasters were especially confirmed by the current COVID-19 pandemic [13–19].

During the second full-scale simulation, staff actions and responses improved. The percentage of required actions performed increased significantly between exercises 1 and 2, and the quality of those actions did as well. Research attempting to prepare pharmacists for humanitarian missions had actually highlighted that simulation was the most effective training because it authentically reproduced their future working environment and readied them for specific situations [39]. Furthermore, the literature also reports that simulation improved teamwork behaviors [40] and enabled a better transfer of acquired competencies to practice [36].

One significant result of the first simulation exercise was that hospital pharmacy chiefs initiated the development of disaster plans (SOPs) if their unit did not have one. The first full-scale simulations raised awareness of the importance of preparedness. Furthermore, they highlighted key points and gaps in preparedness by making disaster situations seem like realistic possibilities, and they showed hospital pharmacy chiefs the potential benefits of being able to implement SOPs. These issues were also underlined in a table-top exercise for hospital pharmacy staff in Australia [41]. Only one of our participating pharmacies already had SOPs for a disaster before the first simulation, but their percentage of required actions taken, and their quality were nevertheless rather similar to those of the other hospital pharmacies. The only significant difference was the time required to move into disaster management mode (time required to gather a disaster management team): this pharmacy was indeed faster. It seems that having a SOP improved reaction times (mainly the time needed to switch to disaster management mode) but without necessarily making staff actions and responses much more efficient in the absence of training. The other pharmacies managed the switch to a disaster management mode much faster during the second full-scale simulation (after having set up SOPs). This underlined that although having SOPs is a precious starting point, it is not enough on its own. More importantly, it is necessary to test and train it [42].

Communication and disaster management were the two hospital pharmacy activities that showed the greatest improvements between the two exercises, but they nevertheless continued to be the two activities with the lowest scores. This is probably because these skills are far more specific to crises than to pharmacy. Other publications have also identified these two activities as weaknesses [43–45]. The classic pharmacy work, even during a disaster, of logistics and clinical pharmacy scored higher than communication or disaster management, probably because those activities are based on staff’s day-to-day knowledge and procedures. One important issue highlighted by the exercise assessors was the separation of flows of disaster-related requests to the pharmacy from routine flows of requests—this required significant improvements. Skills in communication and disaster management were two key points that should certainly be worked on. One option to improve communication would be to train staff in structured communication (mainly restating requests for action to demonstrate comprehension) and integrate it into their day-to-day work. Table-top exercises could be a cheaper means of working on these specific issues [46].

Communication is an essential element of disaster management [47, 48] but it is very often cited as a difficult one to get right [48–51]. Keeping communication effective during a disaster is even more challenging than normal, but it helps enormously in providing an efficient response. Communication gives the direction of information flow and clearly informs staff about their roles in the disaster. Briefings must channel information upwards to inform decision-makers and downwards to ensure implementation [48]. There are several alternative means of communication to facilitate operations in disaster situations, such as the Zello app (a walkie-talkie smartphone application) [47], fixed-line telephones, email, websites, radio announcements, newsletters, or still others [46]. Failure to communicate properly can have significant negative consequences [46], such as anxiety and panic, among staff due to their lack of understanding of the situation [52].

**Fig. 2:**
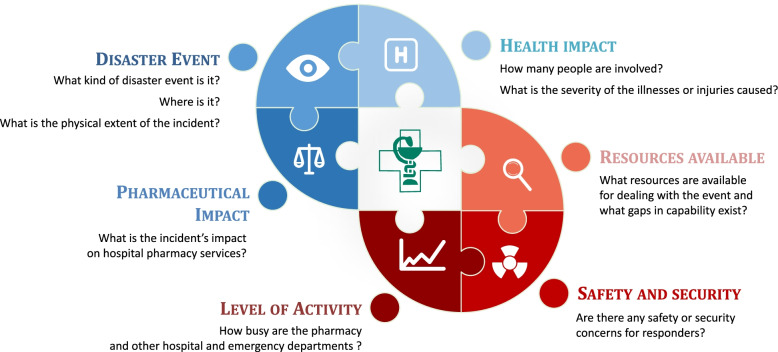
Crisis management, inspired by Fitzgerald G, et al. Disaster health management: A primer for students and practitioners (1^st^ ed.). London: Taylor&Francis; 2016

Another important element is the leadership structure. To be effective in a disaster, chief hospital pharmacists or other leaders should manage and communicate desired outcomes more than the methods or processes used to attain them [48]. A clear, hierarchical, disaster management structure improves communication [46]. In fact, the prehospital settings of major disaster incidents should be coordinated and managed by a specific and appropriate structure in situ [50, 53]. In the hospital setting, the chief pharmacist or his deputy, as that leader, must quickly identify the problem and its healthcare implications and then make adequate decisions, implement management tools, and communicate effectively. Following up on and monitoring the missions and tasks that staff have carried out or still have to carry out within a specific timeframe is essential [8, 53]. The weaknesses in disaster management observed in the present study could have been caused by a lack of awareness and knowledge among hospital pharmacy staff: they may have thought that disaster management was an issue reserved for prehospital disaster locations or emergency departments. The chief pharmacist or his replacement must evaluate and report critical information about the disaster incident. Figure 2 illustrates the key questions that they should ask themselves in response to a disaster in order to ensure appropriate disaster management [48].

Necessary management outputs include defining situational missions and objectives, distributing action plans, giving briefings and issuing situation reports, and optimizing resource use. The disaster management hierarchy should operate vertically and deliver roles and responsibilities[48]. As the literature describes, planning regular briefings to transmit essential information to staff (or team leaders) as a disaster unfolds is highly recommended, as is getting feedback from the field. This information return ensures that the pharmacy chief maintains overall situational awareness. Trained or experienced disaster management team members and regularly updated information and situational dashboards can greatly support disaster leadership [8]. These leadership structures should also be applied in hospital and pharmacy environments, as was shown by the current COVID-19 pandemic [20, 54, 55]. In the present study, SOPs called for and resulted in the use of dashboards in the second simulation, but staff must still become more familiar with using them.

Outside of this study’s framework, one hospital pharmacy performed a third full-scale simulation to determine whether the improvements observed in the second exercise were still valid six months later (data not shown). Indeed, despite the simulation was leaded by a different pharmacist, this final simulation showed that the improvements had been maintained. In addition, the third scenario used focused more on institutional risks (flood, power failure, ventilation failure, etc.) because hospitals must also take into account the types of situations that can happen on their own sites and have immediate consequences on their patients [52, 56, 57]. Therefore, these risks must be considered and prepared for by integrating them into disaster plans, and staff must be trained on how to deal with them [52, 56].

Altogether, these results would suggest to the others healthcare professionals involved in hospital disaster management to include the pharmacy department in their disaster plan and to develop a partnership with it. Indeed, this study has highlighted the importance that such a department has in logistics and clinical drug management during disaster. In this context and in view of the development of this aspect within the pharmacy and to facilitate links with the other hospital departments, it would be useful to identify a respondent for this thematic in each pharmacy.

However, this study has some limitations. First, the number of participating pharmacies was small. It would be useful to expand the number of participants to see whether our results remain similar across the country. Secondly, hospitals C and D experienced the first wave of the COVID-19 pandemic between their first and second full-scale simulations. This may have increased the pharmacy’s awareness and preparedness independently of the drills performed within the framework of our study. Thirdly, although the profiles of the staff at work during the first and second scenarios were quite similar across all the pharmacies, neither their composition nor demographics were recorded, and their initial training levels were not precisely assessed and compared between the two exercises. However, the study’s aim was to evaluate pharmacy preparedness as a whole, not that of the staff themselves. Finally, the evaluation of staff actions and responses was done by human assessors. Evaluations were structured to limit subjectivity, but evaluations can vary and are not always fully reproducible [27].

## Conclusion

This study highlighted the importance of hospital pharmacy disaster preparedness and the value of full-scale simulations. The number of correct actions and responses increased significantly between two simulation exercises. Overall, the full-scale disaster simulations improved the preparedness of the hospital pharmacies and promoted staff awareness. Indeed, the first simulation encouraged hospital pharmacies without a disaster plan to create one and facilitated that task. It appears that training and the existence of standard operating procedures for disaster scenarios helped them to improve their skills and enabled faster implementation of disaster management procedures. Moreover, all the hospital pharmacy teams reported that full-scale simulations were very valuable. Results from further simulations involving these four hospitals and others across Switzerland would be warranted to confirm these preliminary observations in larger-scale setting, as well as possible new training methods such as virtual reality. In the future, other inter-department hospital exercises, including pharmacy, may also be conducted to test disaster plans more widely in the hospital and to disseminate the concept.

## Data Availability

The scenarios were composed of 20 and 24 steps, respectively. The two scenarios will be freely available in French on the Specialised Centre for Emergency and Disaster Pharmacy’s website (http://www.disaster-pharmacy.ch/). Results of evaluations for each full-scale exercises can be asked to the corresponding author.
